# Production of Bioactive Recombinant Reteplase by Virus-Based Transient Expression System in *Nicotiana benthamiana*

**DOI:** 10.3389/fpls.2019.01225

**Published:** 2019-10-08

**Authors:** Ting Ma, Zhiying Li, Sheng Wang

**Affiliations:** ^1^Key Laboratory of Ministry of Education for Protection and Utilization of Special Biological Resources in the Western China, School of Life Science, Ningxia Universisty, Yinchuan, China; ^2^Key Laboratory of Modern Molecular Breeding for Dominant and Special Crops in Ningxia, School of Life Science, Ningxia Universisty, Yinchuan, China

**Keywords:** Reteplase, plant viral expression system, *Nicotiana benthamiana*, transient protein production, Strep-tag II, fibrinolysis activity

## Abstract

To explore a cost-effective alternative method to produce the recombinant thrombolytic drug Reteplase (rPA), a plant viral amplicon-based gene expression system was employed to transiently express bioactive Strep II-tagged recombinant rPA in *Nicotiana benthamiana* leaves *via* agro-infiltration. Several gene expression cassettes were designed, synthesized *in vitro*, and then cloned into *Tobacco mosaic virus* RNA-based overexpression vector. Codon optimization, subcellular targeting, and the effect of attached Strep-tag II were assessed to identify conditions that maximized expression levels of the recombinant rPA in tobacco leaves. We found that codon-optimized rPA with N-terminal Strep-tag II that was aimed to the endoplasmic reticulum as target provided the highest amount of biologically active protein, i.e., up to ∼50 mg from per kilogram fresh weight leaf biomass in less than 1 week. Furthermore, the recombinant rPA was conveniently purified from inoculated leaf extracts by a one-step purification procedure *via* the Strep-tag II. The plant-made rPA was glycosylated with molecular mass of ∼45.0 kDa, and its *in vitro* fibrinolysis activity was equivalent to the commercial available rPA. These results indicate that the plant viral amplicon-based system offers a simple and highly effective approach for cost-effective large-scale production of recombinant rPA.

## Introduction

Thrombotic diseases, especially stroke and heart attack, are the leading life-threatening diseases in the world (Roth and Manwani, 2011). Thrombolytic therapy refers to the use of thrombolytic agents to dissolve or break down blood clots associated with thrombotic disorders (Marder and Sherry, 1988). The commonly used clinical medicine for thrombolytic therapy is tissue plasminogen activator (tPA), a class of serine protease, which converts the zymogen plasminogen to the active enzyme plasmin by means of cleaving the arginine–valine peptide bond, thereby dissolving fibrin clots (Hebert et al., 2016).

tPA is mainly released by endothelial cells (Yau et al., 2015) and is a glycosylated protein containing 527 amino acid residues (72.0 kDa) with 17 disulfide bonds (Baruah et al., 2006). The tPA molecule includes five independently functional domains: the finger domain (residues 4 to 50, F-domain), the epidermal growth factor domain (51 to 87, E-domain), two kringle domains (88 to 176, K1-domain, and 177 to 256, K2-domain), and the serine protease domain (257 to 527, P-domain) (Ny et al., 1984). Hydrolysis of the peptide bond between Arg-275 and Ile-276 of tPA generates two-polypeptide chains, which are linked together by a single interchain disulfide bond (Ny et al., 1984). Various variants of tPA have been developed to improve its clinical efficiency and pharmacokinetic properties.

Reteplase (rPA) is a recombinant form of tPA and is a third-generation thrombolytic agent (Baruah et al., 2006) that was ratified by the Food and Drug Administration in 1996 (Bode et al., 1997). In clinical trials, rPA was proved to possess enhanced thrombolysis capability than what is obtained with tPA (Topol, 1997). It is a truncated mutant of native tPA, consisting of only the K2-domain and P-domain (Gething et al., 1988). The rPA molecule has 355 amino acids (residues 1 to 3 and 177 to 527) of the 527 amino acids of native tPA with two N-linked oligosaccharides at Asn-184 and -448 (Noble and McTavish, 1996). rPA is commercially produced in *Escherichia coli* where it accumulates in inclusion bodies and thus needs to be renatured *in vitro* to restore biological activity (Fathi-Roudsari et al., 2018). Since the process is grossly laborious and inefficient, the commercially available rPA is very expensive. This high pricing has limited the application of the recombinant rPA, especially in the developing and third-world countries. To overcome this issue, alternative production systems have been tested: Chinese hamster ovary cells (Davami et al., 2010), insect cells (Aflakiyan et al., 2013), yeast (Shu-Guang et al., 2006), transgenic animals (He et al., 2018), and transgenic plants (Zhang et al., 2008; Nasab et al., 2016; Hidalgo et al., 2017). However, most of these were not found to be appropriate.

Plants have recently been regarded as an excellent alternative for producing recombinant protein (Lico et al., 2012). Plant platform offers numerous potential advantages over the traditionally used non-plant expression systems, including low-capital equipment, low-energy requirements, easy scale-up, reduced risk of carrying pathogen contamination, and ability to posttranslational modifications (Obembe et al., 2011). The production of recombinant proteins in plants can be achieved by either stable or transient expression (Streatfield, 2007). Stable transformation is often time consuming, whereas transient expression can be very fast, and the yields of the protein of interest are generally higher (Xu et al., 2012). Plant virus gene vectors are currently used in plant molecular farming, and agro-infiltration is an effective strategy for the delivery of viral vectors to their host plants (Peyret and Lomonossoff, 2015). An outstanding advantage of virus-based expression system is that the target proteins can be produced at very high levels in a matter of days due to viral amplification (Hefferon, 2017). Among various plant viral gene vectors, *Tobacco mosaic virus* (TMV)-based expression vector has been widely and successfully applied to express biologically active recombinant proteins (Brewer et al., 2018).

In the study, we developed a strategy to produce enzymatically active recombinant rPA in *Nicotiana* plants by viral expression vector. Several gene expression cassettes were designed to improve the accumulation level of recombinant rPA in *Nicotiana benthamiana* leaves. All expression cassettes were synthesized *in vitro* and then cloned into a TMV-based gene expression vector (TRBO) (Lindbo, 2007). Viral vectors carrying each cassette were then transformed into *A. tumefaciens* independently and co-inoculated into *N. benthamiana* leaves with another construct expressing the P19 silencing suppressor protein of *Tomato bushy stunt virus* (TBSV). The effects of codon optimization, subcellular targeting, and the position of Strep-tag II on the expression of recombinant rPA were examined. A one-step purification procedure utilizing Strep-tag II affinity chromatography was established. The *in vitro* fibrinolysis activity of plant-produced rPA purified from whole leaf homogenates was assessed.

## Materials and Methods

### Plant Materials and Growth Conditions

*N. benthamiana* plants were grown in small pots (5 inches in diameter) with autoclaved soils under controlled growth conditions (22–25°C, 65% relative humidity, 8/16-h dark/light cycle). All plants were supplemented with water and Hoagland solution when required.

### Construction of Gene Expression Cassettes

The *rPA* gene sequence lacking its native signal peptide (SP) was codon-optimized *in silico* (Invitrogen, Beijing, China) to facilitate expression in *N. benthamiana*. Two different codon-optimized versions of *rPA* gene were obtained. One codon-optimized sequence was based on the characteristics of *N. benthamiana* codon usage bias, while the other was derived from *N. tabacum*. In all expression cassettes, the native SP of *rPA* was substituted by an 87-base pair (bp) sequence coding for the tobacco pathogenesis-related protein 1b (Pr1b) SP (Gene bank: D90197.1). An endoplasmic reticulum (ER) retention SP (KDEL) was placed at the C-terminal end of rPA sequences for targeting the recombinant rPA to ER in most cassettes except of one cassette designed to target rPA to the apoplastic space. The Strep-tag II sequence (WSHPQFEK) was inserted between rPA sequences and the ER SP in most of the cassettes or between the Pr1b SP and rPA sequences in one cassette. All gene expression cassettes were synthesized *in vitro* (GenScript, Nanjing, Jiangsu, China). Additionally, the synthesized sequences contain *Pac* I and *Not* I restriction sites on each prime end for cloning procedures.

### Construction of Viral Vector Expression Systems

The TMV-based vector (pJL TRBO-G) (Lindbo, 2007) was used to transcribe the different rPA expression cassettes. The plasmids with each cassette and the pJL TRBO-G vector were double digested with *Pac* I and *Not* I, respectively. The individual inserts were then ligated to the pJL TRBO-G vector yielding viral expression vectors. The recombinant plasmids were transformed into competent cells of *E. coli* DH5α (TransGenBiotech, Beijing, China) according to the manufacturer’s specifications. After screening by colony polymerase chain reaction, the positive plasmids were retransformed into *Agrobacterium tumefaciens* GV3101 as described earlier (Shen and Forde, 1989).

### Agro-Infiltration and Incubation Of *N. benthamiana* Plants

*A. tumefaciens* cells, having TRBO expression cassettes or pCBNoX p19, were cultivated in 4-ml Luria–Bertani mediums (containing kanamycin, rifampicin, and gentamycin, 50 µg/ml for each) at 28°C with shaking (250 rpm). After 24 h of the cultivation, the cultures were transferred into 100-ml Luria–Bertani mediums (supplemented with 200-μM acetosyringone) and were grown overnight under the same cultivation condition. Afterward, cells were centrifuged for 10 min at 4,000×*g* and resuspended in 10-mM 2-(N-morpholino)ethanesulfonic acid buffer (pH 5.6, 10-mM magnesium chloride and 200-μM acetosyringone) to achieve an appropriate density. The bacterial suspension with individual TRBO expression cassettes were mixed with that of pCBNoX p19 (a vector for expressing TBSV-P19 gene silencing suppressor) in equal proportions and were then left in a dark place at 25°C for 2–3-h incubation. The incubated mixture was infiltrated into the abaxial surface of *N. benthamiana* leaves by a needleless 1-ml syringe. The agro-infiltrated plants were put into the growth chamber and were grown for another 7–12 days. The infiltrated leaves were sampled on date and stored at -80°C for subsequent analysis.

### Extracting Reteplase From Agro-Infiltrated *N. benthamiana* Leaves

The harvested leaves were grinded to a very fine powder in liquid nitrogen, and then, the extraction buffer (50-mM Tris-hydrochloride, pH 8.0, 100-mM sodium chloride, 5-mM ethylenediaminetetraacetic acid, 0.1% Tween 20, 0.1% protease inhibitors cocktail) was added into the leaf powder at a ratio of 1:4 (g/ml). The homogenate was rested at 4°C for about 1 h. After refrigerated centrifugation at 12,000×*g* for 15 min, the supernatant containing the total soluble proteins (TSPs) was recovered for further analysis.

### Sodium Dodecyl Sulfate-Polyacrylamide Gel Electrophoresis and Western Blot Analysis

Generally, 15 μl of TSPs for each sample was loaded on 12% sodium dodecyl sulfate-polyacrylamide gel electrophoresis (SDS-PAGE) gels under reducing conditions. To confirm that the nontarget bands were due to proteolysis-mediated cleavage of rPA, the nonreducing SDS-PAGE gels were employed. Electrophoresis was performed for about 1 h at 180 V, and then, one of the gels was stained in Coomassie Brilliant Blue G-250 or sliver; another one was transferred to an NC membrane for 2 h at 80 mA. The membrane was blocked with 5% nonfat dry milk in phosphate-buffered saline (PBS) overnight, washed three times with 1× PBS with Tween 20 buffer at room temperature for 5 min each. The blots were developed with either 1:5,000 dilution of rabbit polyclonal anti-human tPA antibody or with 1:1,000 rabbit polyclonal anti-strep tag II antibody followed by 1:5,000 goat anti-rabbit horseradish peroxidase-conjugated secondary antibody. Enhanced chemiluminescence solution for Western blot (GE Healthcare, USA) was used to detect the specific immunoreactive proteins. In order to determine the fold changes in recombinant rPA expression before/after the optimization, the Western blot images were then analyzed by densitometric method using Quantity One 4.62 (Bio-Rad) (Taylor et al., 2013). The large RuBisCO subunit (Rbc L) stained with Coomassie Blue was used as loading controls for this quantification. The data were collected from three separate experiments, and mean values were performed with Student’s *t*-test.

### Quantification of Plant-Derived Reteplase

Indirect enzyme-linked immunosorbent assay (ELISA) was employed to estimate the amount of rPA in the infiltrated *N. benthamiana* leaves. Briefly, an ELISA plate was coated with 100 μl of TSPs and incubated overnight at 4°C. The plate was then washed three times with 300 μl of PBS per well at 5-min intervals. The anti-tPA rabbit polyclonal antibody (1:10,000 dilution) in PBS was added to each well and then rested at 37°C for 2 h. Followed by another three times washing, goat anti-rabbit horseradish peroxidase-conjugated secondary antibody (1:5,000 dilution) was added into each well and incubated for 2 h at 37°C. After a new round of washing, 100 μl of tetramethylbenzidine was added into each well, and the plate was incubated for 15–30 min. After that, 100 μl of phosphoric acid (1.0 M) per well was pipetted to stop the enzyme reaction. The absorbance was read by a microplate reader at 450 nm. A standard curve of rPA was simultaneously established to calculate the relative accumulation of rPA in the leaves. The samples were collected from three independent agroinoculation experiments, and the measurement was repeated three times for each sample.

### Purification of Reteplase From Total Leaf Homogenates

The agro-infiltrated leaves with pJL-Pr1b-rPA NSK were used to purify recombinant rPA from total leaf homogenate. The leaf tissues were ground in liquid nitrogen, and then, the binding buffer (100-mM Tris-hydrochloride, 150-mM sodium chloride, 1-mM ethylenediaminetetraacetic acid, 100 μg/ml avidin, 0.1% protease inhibitors cocktail, pH 8.0) was added at a ratio of 1:4 (g/ml). The crude extracts were centrifuged at 12,000×*g* for 15 min at 4°C. The supernatant was filtered through a 0.22-μm filter (Millipore) and, then, was loaded on StrepTrap™ HP, affinity gel (GE). The target protein was captured and eluted by elution buffer (2.5-mM desthiobiotin in binding buffer) according to the manufacturer’s specifications.

### Endoglycosidase H and N-Glycosidase F Digestions

The purified rPA protein was deglycosylated with Endo H or PNGase F (New England Biolabs) for 2 h at 37°C following the manufacturer’s instructions. The purified rPA without adding glycosidase treated in the same conditions and the *E. coli*-produced rPA (unglycosylated) was taken as controls. All samples were then analyzed by Western blot-based detection using the anti-tPA rabbit polyclonal antibody.

### *In Vitro* Activity Assay of Plant-Derived Reteplase Protein

A modified fibrin agarose plate assay (Astrup and Müllertz, 1952) was used to estimate the plasminogen activation of plant-derived rPA. In brief, 20 ml of 0.8% agarose gels solution was prepared in normal saline and kept at 55°C to avoid any local gelling. Subsequently, human fibrinogen (13.2 mg), plasminogen (140.0 g), and thrombin (1.4 IU) were added into the gels solution one by one with shaking. The mixture was then poured into a plastic Petri dish (8 cm in diameter) and incubated at 25°C for at least 30 min. The purified rPA in different concentration with or without plasminogen activator inhibitor-1 (1.0 μg) was placed for 20 min at 25°C. *E. coli* expressed rPA protein was used as a positive control. All of the samples were then spotted on the fibrin-coated plate. After overnight incubation at 37°C, fibrin clearance was visualized by brief staining with Coomassie Blue.

## Results and Discussion

### Description of *Tobacco Mosaic Virus*-Derived Vectors for Transient Expression of Reteplase in *N. benthamiana*

**Figure 1:**
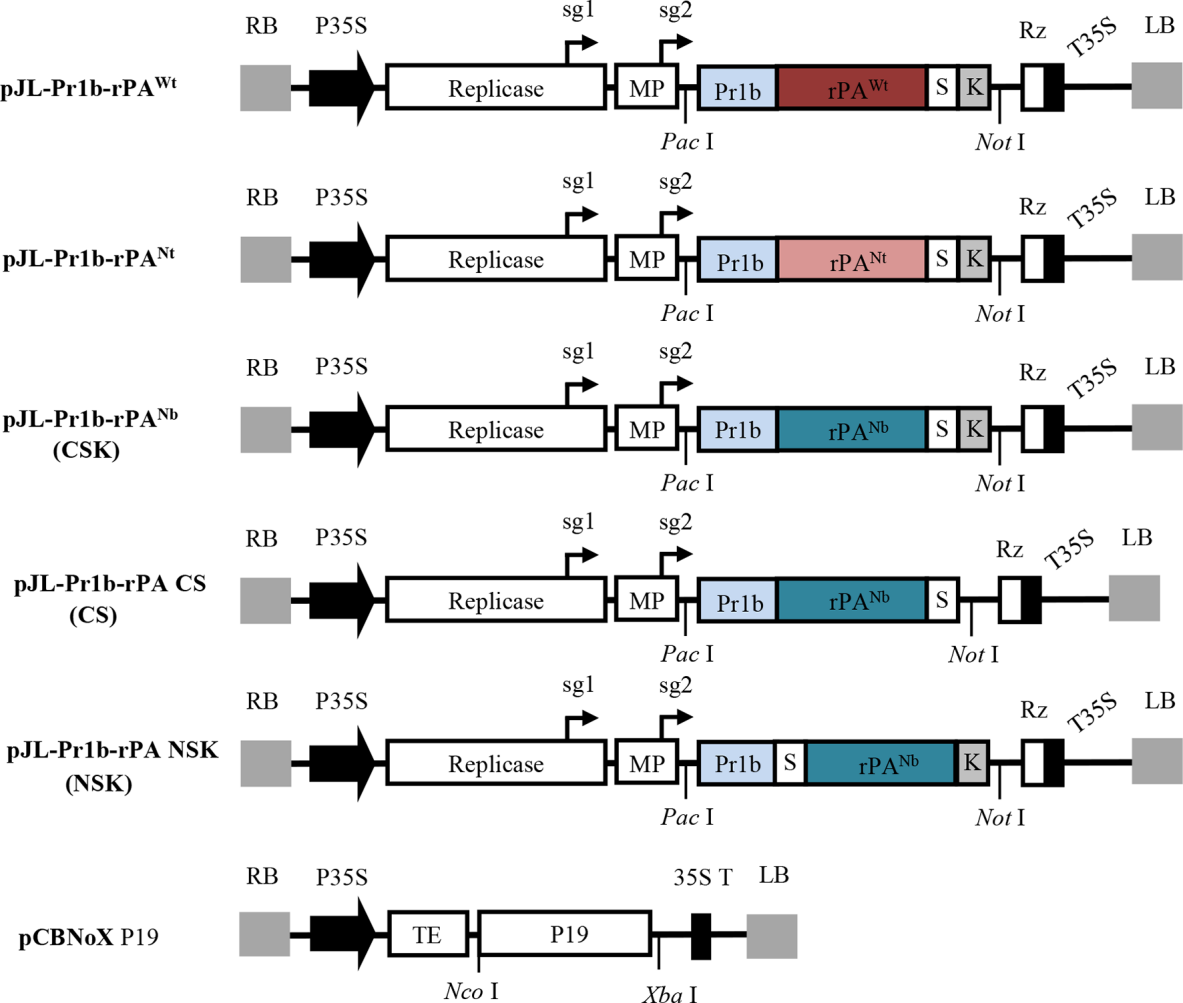
Schematic representation showing the T-DNA region of the various genetic constructs expressed in this study. LB and RB, the left and right T-DNA borders; P35S, enhanced *Cauliflower mosaic virus* 35S promoter; Replicase, RNA-dependent RNA polymerase of TMV; sg1 and sg2, subgenomic mRNA1 and mRNA2 promotors of the TMV; MP, movement-protein of TMV; Pr1b, Pr1b tobacco secretory SP; rPA^wt^, the wild-type sequence of rPA; rPA^Nt^ and rPA^Nb^, the *N. tabacum* and *N. benthamiana* codon-optimized rPA genes; S, the strep-tag II; K, ER retention signal; Rz, ribozyme; T35S, CaMV transcription terminator; TE, tCUP translational enhancer; p19, gene of silencing suppressor p19 from TBSV; *Pac* I, *Not* I, *Nco* I and *Xba* I, restriction enzymes. Note that boxes are not drawn to scale.

To produce the recombinant rPA in *N. benthamiana* plants, different gene expression cassettes were designed and are shown in **Figure 1**. The 1,065-bp fragment encoding the truncated version of human tPA was cloned into pJL TRBO-G vector. pJL TRBO-G is a TMV-based replicon that lacks most of its coat protein gene and was modified to allow for facile insertion of genes of interest. Accordingly, this design allows for efficient foreign gene expression in *N. benthamiana* during replication of the viral vector (**Figure 1**).

In most cases, sequence modifications are usually required before the foreign genes can be optimally expressed in plants. Since rPA is a glycosylated protein, an ER-targeting plant SP had to be added to guide the recombinant protein through the secretory pathway. SignalP 4.1 Server (at http://www.cbs.dtu.dk/services/SignalP/), an online tool for predicting SP cleavage sites and efficiencies, was employed to identify a plant-compatible SP for the rPA gene. The tobacco Pr1b SP was selected based on predicted accurate and efficient processing (data not shown). Consequently, an 87-bp sequence coding for the tobacco Pr1b SP was placed at the N-terminal end of the rPA gene.

To obtain high-expression levels of rPA, the rPA gene sequence was codon-optimized based on the characteristic codon usage bias of either *N. benthamiana* or *N. tabacum*. After codon optimization, the sequences were analyzed for possible intron splice sites by using NetPlantGene Server (at http://www.cbs.dtu.dk/services/NetPGene/) so as to avoid unwanted spliceosome processing in the cell nucleus. Additionally, a KDEL motif was placed at the C-terminal end of rPA sequence to allow for retention of recombinant proteins in the ER. Finally, an affinity tag, Strep-tag II, was inserted between rPA sequence and the C-terminal KDEL motif to use in downstream protein purification. Two tobacco-optimized and one unmodified versions of the rPA expression cassettes were synthesized *in vitro* and subcloned into pJL TRBO-G vector. Three TMV-derived vectors, pJL-Pr1b-rPA^Wt^ carrying unmodified rPA gene, pJL-Pr1b-rPA^Nt^, and pJL-Pr1b-rPA^Nb^ with codon-optimized rPA genes, were obtained.

All three expression vectors allow targeting the recombinant rPA proteins to the ER. In order to investigate the effects of cell-compartment targeting on the expression of rPA in *N. benthamiana* plant leaves, another expression construct, pJL-Pr1b-rPA^Nb^ (CS), was designed and constructed to target the recombinant protein to the apoplastic compartment. Unlike the ER-targeting constructs pJL-Pr1b-rPA^Nb^ (CSK), the C-terminal KDEL motif in pJL-Pr1b-rPA^Nb^ CS was removed.

Fusion tags are commonly used to purify the recombinant proteins and can be added at either terminus of a target protein (Terpe, 2003). The position of fusion tag can significantly influence the properties of its fusion protein partners, such as their accumulation and physicochemical behaviors in solution (Mohanty and Wiener, 2004; Xu et al., 2008). Strep-tag II was used in our study and was originally placed at C-terminus of rPA [pJL-Pr1b-rPA^Nb^ (CSK)]. To determine the effect of the Strep-tag II position on rPA expression level in *N. benthamiana* plant, another expression vector pJL-Pr1b-rPA (NSK) was constructed with the Strep-tag II at the N-terminus of the rPA gene.

All constructs were transformed into *A. tumefaciens* GV3101, and *Agrobacterium*-mediated transient expression was employed for conveniently testing the accumulation level of recombinant rPA in *N. benthamiana*. To inhibit the posttranscriptional gene silencing in plants, a construct expressing TBSV-P19 gene silencing suppressor (Saxena et al., 2011) was co-inoculated along with the individual TRBO expression vectors (**Figure 1**).

### Transient Expression of Reteplase in *N. benthamiana* Leaves

The density of the *Agrobacterium* suspension was important for the expression levels of foreign gene, and suspensions with high densities [above optical density 600 (OD_600_) = 1.0] often cause yellowing or wilting on the infiltrated leaf tissue (Kagale et al., 2012). Therefore, the analyses were performed with densities of *Agrobacterium* suspensions at OD_600_ = 0.4 or 0.8.

**Figure 2:**
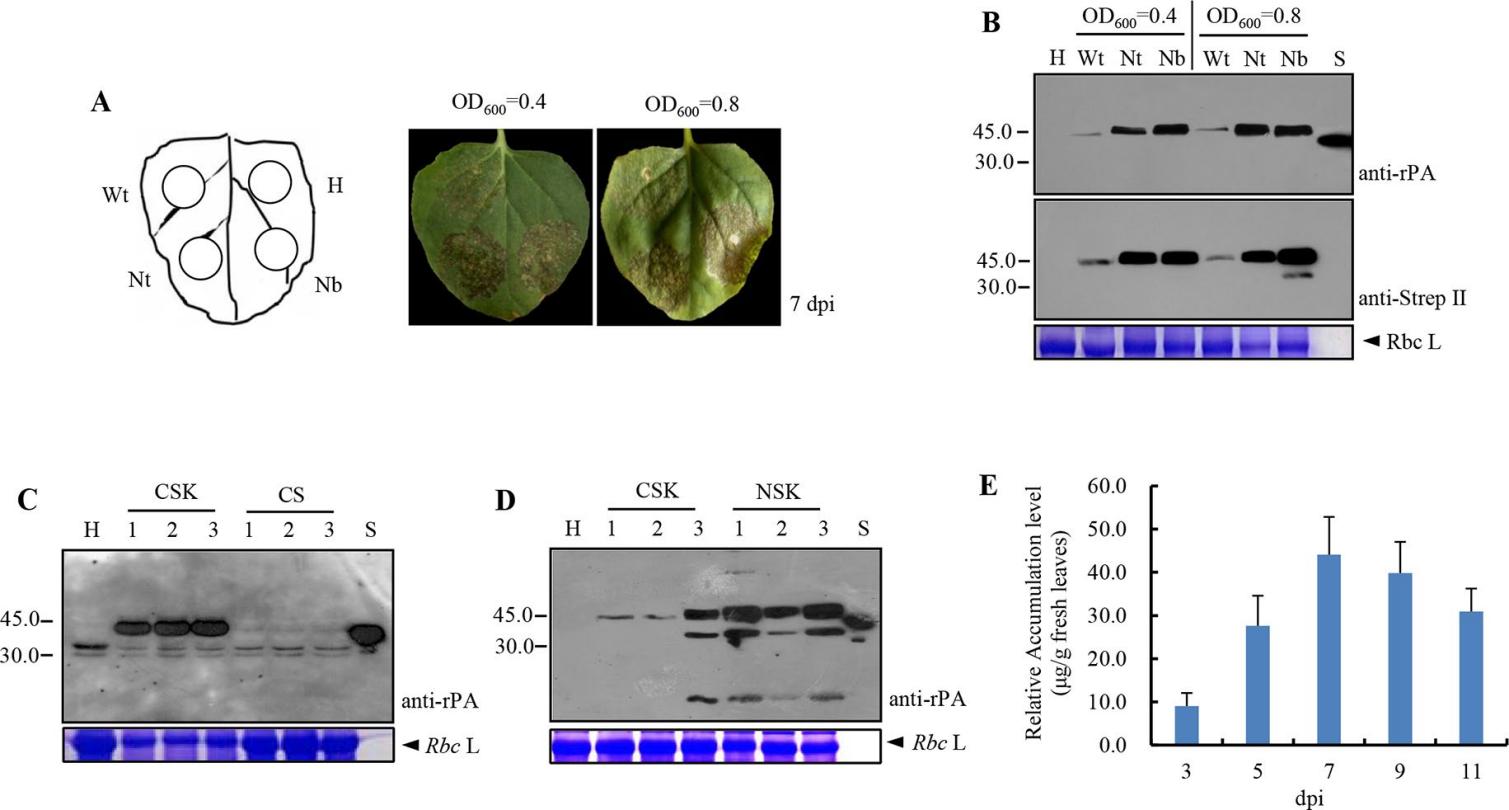
Analysis of recombinant Reteplase (rPA) accumulation in *N. benthamiana*. The individual plasmids were transformed into *A. tumefaciens* and co-infiltrated with *Agrobacterium* carrying pCBNoX p19 constructs in equal amounts into *N. benthamiana* leaves. **(A)** Necrosis appeared in the infiltrated area with two concentrations of *Agrobacterium* inocula. The codon-optimized and non-optimized constructs were infiltrated at different areas within a leaf (left panel), and photographs were taken at 7 days post-inoculation (dpi) (right panel). Wt, codon non-optimized construct, pJL-Pr1b-rPA^Wt^; H, non-inoculated leaf, negative control; Nt, *N. tabacum* codon-optimized construct, pJL-Pr1b-rPA^Nt^; Nb, *N. benthamiana* codon-optimized construct, pJL-Pr1b-rPA^Nb^; OD_600_ = 0.4 and OD6_600_ = 0.8, the concentration of *Agrobacterium* inoculum, measured by spectrophotometer at the wavelength of 600 nm. **(B)** The codon-optimized constructs displays stronger protein accumulation. Samples were collected from the agro-infiltrated zones of *N. benthamiana* leaves at 7 dpi and TSPs isolated for Western blot analysis. H, Wt, Nt, and Nb, as is in (A); S, rPA produced in *E. coli* (Roche), positive control; anti-rPA, rabbit polyclonal to human tissue-type plasminogen activator (tPA) (Abcam); anti-Strep tag II, rabbit polyclonal to Strep-tag II (GenScript); Rbc L, the large subunit of Rubisco stained with coomassie brilliant blue, protein loading control. Protein molecular weight markers in kDa are shown on the left. **(C)** ER retention signal increases rPA protein accumulation. Leaf sectors were infiltrated with *Agrobacterium* containing pJL-Pr1b-rPA^Nb^ (CSK) for targeting the protein into the ER, pJL-Pr1b-rPA CS (CS) for targeting in the apoplast (AP). Samples were harvested at 7 dpi, and protein samples were subjected to Western blot for analysis of the presence of the rPA protein. H, non-inoculated leaf, negative control; lanes 1, 2, and 3, TSP extracts of three leaves transfected independently; CSK, ER-targeted rPA protein; CS, AP-targeted rPA protein; S, anti-rPA and Rbc L, as is in (B). Protein molecular weight markers are shown on the left. **(D)** N-terminal strep-tag II enhances rPA production. The N-terminal (pJL-Pr1b-rPA NSK) and C-terminal strep-tag II constructs (pJL-Pr1b-rPA^Nb^, CSK) were infiltrated into *N. benthamiana* leaves, and samples were harvested at 7 dpi. The presence of the rPA protein was shown by Western blot analysis. H, non-inoculated leaf, negative control; lanes 1, 2, and 3, TSP extracts of three leaves transfected independently; CSK, rPA with C-terminal Strep-tag II; NSK, rPA with N-terminal Strep-tag II; S, Reteplase (rPA) produced in *E. coli* (Roche), positive control; anti-rPA, rabbit polyclonal to human tissue-type plasminogen activator (tPA) (Abcam); anti-strep tag II, rabbit polyclonal to strep-tag II (GenScript); Rbc L, the large subunit of Rubisco stained with Coomassie Brilliant Blue, protein loading control. Protein molecular weight markers in kDa are shown on the left. **(E)** Time course for accumulation of recombinant rPA protein in *N. benthamiana* plants infected with pJL-Pr1b-rPA NSK. Leaves infected with pJL-Pr1b-rPA NSK were harvested at 5–11 dpi. The un-inoculated plant sample was used as the control sample. Protein samples were subjected to indirect ELISA for analysis of recombinant rPA protein level in crude protein samples. The recombinant rPA protein concentration in plant samples was determined by extrapolation of the standard curve prepared from a known concentration of the *E. coli* recombinant rPA protein mixed with the un-inoculated plant protein. The data represent the average of three independent measurements ± SD.

To explore the effects of codon optimization on the amount of recombinant rPA produced in plants, the mixes of *Agrobacterium* cultures, which contain one of the TRBO expression cassettes (pJL-Pr1b-rPA^Wt^, pJL-Pr1b-rPA^Nt^, and pJL-Pr1b-rPA^Nb^) and pCBNoX p19, infiltrated into the leaves of *N. benthamiana*. Five to 7 days after infiltration, in all cases, necrosis appeared in the infiltrated area under both densities of *Agrobacterium* mixes. Twelve days after infiltration, the tissue in the infiltrated area was completely dried. Necrosis with pJL-Pr1b-rPA^Wt^ (Sector Wt) was weaker than that with pJL-Pr1b-rPA^Nt^ (Sector Nt) or pJL-Pr1b-rPA^Nb^ (Sector Nb). In contrast, the un-infiltrated areas (Sector H) did not show any sign of necrosis (**Figure 2A**). In addition, the areas infiltrated alone with empty *Agrobacterium* cultures or *Agrobacterium* carrying pCBNoX p19 was still kept healthy (data not shown). Similarly, TMV-based expression vectors led to complete leaf necrosis in the transient expression of human growth hormone (Gils et al., 2005), hepatitis B core antigen (Huang et al., 2006; Huang et al., 2008), mAbs of the IgG (Giritch et al., 2006), tuberculosis ESAT6 antigen (Dorokhov et al., 2007), actinohivin (Matoba et al., 2010), human epithelial mucin1 (Pinkhasov et al., 2011), human complement factor 5a (Nausch et al., 2012), or envelope protein of *West Nile virus* (He et al., 2014). However, leaves agro-infiltrated with pJL TRBO-G vector remained healthy at 14 days post-inoculation (dpi), and the expression level of green fluorescent protein in infiltrated leaves is much higher than that of the previously mentioned recombinant proteins. Pinkhasov et al. (2011) speculated that the plant metabolism might be interfered by the expressed protein, thereby resulting in phenotype alteration or cell death. However, no necrosis of tissue was observed when rPA was expressed at the amount of 0.62 μg/g fresh leaf tissue *via Zucchini yellow mosaic virus* (ZYMV)-based vectors (Javaran et al., 2017). We estimated that the amount of rPA produced by the ZYMV-derived vector was close to what was obtained with the non-optimized pJL-Pr1b-rPA^Wt^ vector. Nevertheless, the reason for the observed rPA-induced leaf necrosis remains unknown currently.

Subsequently, the expression of rPA in infiltrated areas was measured at 7 days after infiltration by Western blot with rPA produced by bacteria as a positive control. The predicted molecular mass of intact rPA without SP is about 39.0 kDa, and it contains two N-glycosylation sites (Noble and McTavish, 1996), indicating that the protein of approximately 45.0 kDa represents the intact mature glycoprotein. Western blot analysis with both anti-rPA antibody and antiserum against Strep-tag II showed clear signals at the expected position corresponding to 45.0 kDa (**Figure 2B**). Interestingly, intact rPA was still present in necrotic leaf tissues. There was no difference in the relative accumulation of rPA for both densities of *Agrobacterium* suspensions used for infiltration in the three treatments. High-level replication of TMV may explain this observed phenomenon. Therefore, the lower densities of *A. tumefaciens* suspensions (OD_600_ = 0.4) were used in all subsequent experiments. Notably, different levels of rPA protein expression were found between the tobacco plants infiltrated by pJL-Pr1b-rPA^Wt^ (carrying the wild-type *rPA* gene) and pJL-Pr1b-rPA^Nt^ or pJL-Pr1b-rPA^Nb^ (carrying the codon-optimized *rPA* gene). Densitometric analysis of the specific rPA immunoblot bands revealed ∼15-fold increases with the codon-optimized expression cassettes while comparing with the non-optimized one (**Figure 2B**). This result suggests that codon optimization is very effective for high-level expression of rPA protein in plants. However, there was no effect on rPA protein accumulation between two codon-optimized vectors, pJL-Pr1b-rPA^Nt^ and pJL-Pr1b-rPA^Nb^ (**Figure 2B**). We speculate that the characteristics of codon usage bias between *N. tabacum* and *N. benthamiana* are similar.

To determine whether subcellular targeting could influence rPA expression in tobacco leaves, CSK and CS vectors, by which the recombinant rPA was targeted to the ER or the apoplast, respectively, were agro-infiltrated in *N. benthamiana* leaves. Necrosis appeared in the CSK vector infiltrated area instead of CS. In order to determine whether the differences in symptoms were due to differences in rPA expression, we analyzed tissue extracts from agro-infiltrated leaves by Western blot at 7 days after infiltration using rPA antibodies. The rPA protein targeted to the apoplast was undetectable in immunoblot analyses (**Figure 2C**). Instead, the ER-targeted rPA protein with predicted molecular weight was clearly visualized (**Figure 2C**). The non-detectable amount of rPA accumulated in the apoplast may explain the absence of symptoms in CS-infiltrated leaves. The result indicated that ER localization allowed recombinant rPA to accumulate much higher levels.

Since the position of tags might affect the yield of the recombinant proteins, we explored the effect of the positions of Strep-tag II on the accumulation of the rPA protein. TSPs were extracted from three leaves independently transfected with either CSK (tag at C-terminus) or NSK (tag at N-terminus) vector, and the *E. coli*-produced rPA was used as a positive control. The presence of the rPA protein was detected at 7 days after inoculation with rPA antibody by Western blot analysis. There was no immune cross-reactivity with the TSPs extracted from non-inoculated leaves, and the signals with position corresponding to 45.0 kDa were shown in both CSK and NSK-inoculated treatments. Clearly, the accumulation of recombinant rPA was greatly influenced by the tag position, and the amount of rPA with Strep-tag II at the N-terminus was higher than the one with tag at the C-terminus (**Figure 2D**). In addition to the 45.0-kDa major band corresponding to intact rPA protein, bands around 31.0 and 14.0 kDa were also present (**Figure 2D**). This is expected since rPA can be cleaved into two fragments by proteolysis (Ny et al., 1984).

The expression of rPA in NSK-infiltrated areas was quantified using rPA ELISA. The recombinant rPA attained its maximal value of about 46.0 μg/g fresh tissue at 7 days after inoculation (**Figure 2E**). Over the past decade, the bioactive rPA was expressed stably in transgenic seaweed (Zhang et al., 2008), transplastomic tobacco plants (Nasab et al., 2016), and transplastomic tobacco cell cultures (Hidalgo et al., 2017) or was expressed transiently in cucurbit plants by using ZYMV-based expression vector (Javaran et al., 2017). The level of the expression in the transgenic seaweed or ZYMV-infected cucurbit plants is not promising. However, it is notable that the accumulation of rPA in transplastomic tobacco plants was as high as approximately 300.0 µg/g fresh leaf tissue (Nasab et al., 2016). Nevertheless, one of the outstanding benefits of the transient expression system is that it was able to extremely rapidly express the recombinant protein. Although the expression level of rPA in our study is lower than that obtained from the transplastomic tobacco plants, our approach have an inherent potential to be used as a convenient expression platform for further improving clinical efficiency and pharmacokinetic properties of rPA by a genetically engineering approach.

For literatures related to rPA production in *E. coli* systems, some studies have not been published, and the details of some experimental methods are not available (Mohammadi et al., 2019). Therefore, it is hard to tentatively compare the potential costs of rPA production between *E. coli*-based commercial system and plant expression platform. However, it has been estimated that purification and downstream processing of recombinant proteins represents 80–90% of the cost of producing pharmaceuticals (Sabalza et al., 2014). In terms of the yield, the plant-produced rPA may not be comparable with that obtained from *E. coli* yet. Nevertheless, the recombinant rPA produced in *E. coli* mostly accumulates in inclusion bodies and thus needs to be renatured to restore biological activity (Mohammadi et al., 2019). Therefore, the soluble form of plant-produced rPA avoids this tedious and inefficient process that should be done for *E. coli*-expressed rPA. Theoretically, the production of rPA in plant may possess a good benefit for the cost reduction of the final rPA product.

### Purification of Reteplase Expressed in *N. benthamiana* Leaves

Purification of rPA protein was carried out from 5.0 g of agro-infiltrated *N. benthamiana* leaves by Strep-tag II affinity chromatography. Leaves agro-infiltrated with NSK were sampled at 7 days after inoculation and, then, were mechanically homogenized. In order to overcome unwanted proteolysis during the process of extraction and purification, a cocktail of protease inhibitors was added into the extraction buffer. In addition, since biotin in the extract may interfere with the subsequent affinity chromatography (Witte et al., 2004), crude extracts were mixed with an amount of avidin to avoid the irreversible binding of free biotin.

**Figure 3:**
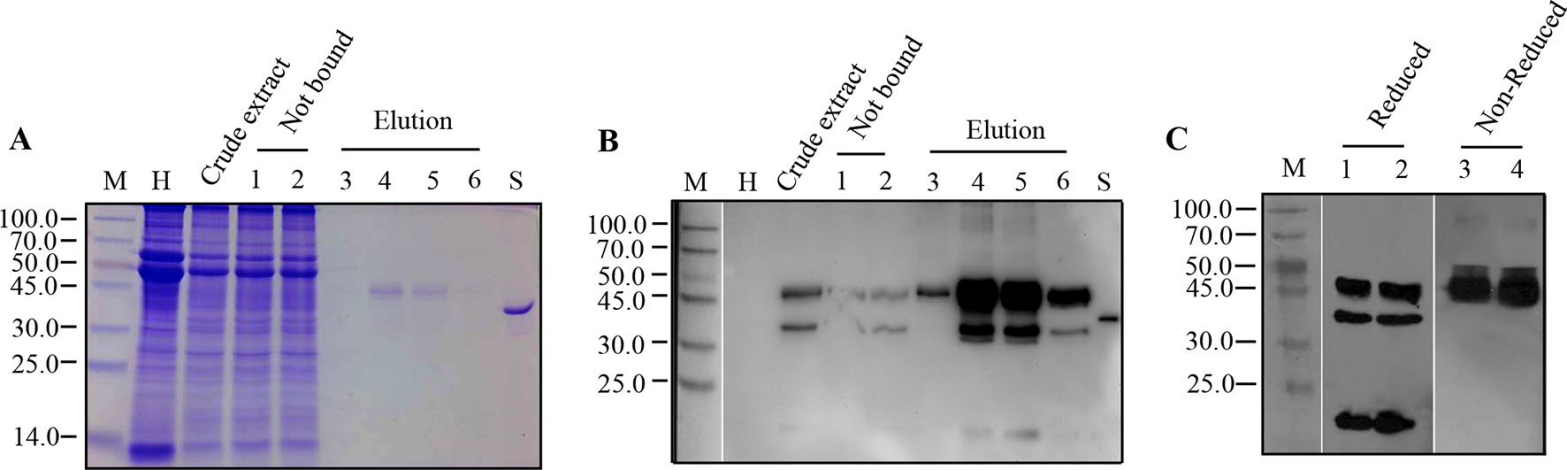
Purification of recombinant Reteplase (rPA) produced in *N. benthamiana*. rPA was purified from leaves infiltrated with the N-terminally strep II-tagged and ER-targeted rPA construct pJL-Pr1b-rPA NSK. Leaves were harvested at 7 dpi, and rPA from leaves was extracted for affinity chromatography using a StrepTrap HP (1 ml) column (GE). **(A)** Purification of rPA visualized by Coomassie Blue-stained polyacrylamide gel. M, Protein molecular weight markers in kDa; H, total soluble protein extract from control non-inoculated leaf; Crude extract, extract of total soluble proteins from pJL-Pr1b-rPA NSK inoculated leaf; Not bound, proteins not bound after incubation with StrepTrap HP, repeats (lanes 1 and 2); Elution, the tagged protein eluted with biotin, different fractions from the elution peak (lanes 3, 4, 5, and 6); S, Reteplase (rPA) produced in E. coli (Roche), positive control. **(B)** Western blot showing purification of rPA. Antibodies against tPA were used in Western blot. Abbreviations are explained as in **(A)**. **(C)** Immunoblot analysis of Strep-tag II purified rPA under reduced and non-reduced conditions. Purified rPA protein was separated on 10% polyacrylamide gel under reduced and non-reduced conditions independently and transferred to 0.45-μm NC membranes. The blot was probed with polyclonal antibody against tPA. M, Protein molecular weight markers in kDa; lanes 1 and 2, repeats of purified rPA protein under reduced condition; lanes 3 and 4, repeats of purified rPA protein under non-reduced condition.

A single peak was present in the affinity chromatography after elution (data not shown). Samples from each fraction, such as the crude extract, flow through, and the elution, were visualized by Coomassie-stained SDS-PAGE and, then, detected by Western blot using rPA antibodies. A 45.0-kDa protein band representing intact rPA was observed in both Coomassie-stained gels (**Figure 3A**) and Western blots (**Figure 3B**) in the elution fractions, demonstrating the successful purification of the Strep-tagged rPA. Also, a faint 45.0-kDa band was also detected in the flow-through fraction (**Figure 3B**), suggesting that there is still room for optimization of the purification procedure. Related issues, i.e., buffer/sample composition, column capacity, extra protein linker, and the biotin content, should be considered systematically and integrally. Regardless, the Strep-tag II offers a considerably easier and faster strategy for one-step purification of recombinant rPA from *N. benthaniana* leaf extracts.

After purification *via* Strep-tag II, the recombinant rPA was concentrated and desalted using centrifugal ultrafiltration. The concentrated rPA protein was then analyzed by Western blot under reduced or non-reduced condition to confirm that the other two smaller extra bands were indeed cleavage products of intact recombinant rPA. Reduced samples showed three bands with position corresponding to 45.0 kDa, followed by 31.0 kDa and 14.0 kDa (**Figure 3C**). Conversely, non-reduced sample gave rise to a single band with position corresponding to approximately 45.0 kDa (**Figure 3C**), demonstrating that the recombinant rPA protein was cleaved by proteolysis. However, it is unclear if the cleavage occurred inside plant cells or during extraction and purification process. Protease inhibitors were added during the extraction and purification, and cleavage products were also detected in immunoblots of the crude extract, suggesting that cleavage may have occurred inside plant cells. Similarly, cleavage products from foreign proteins, such as antibody, equistatin, human growth hormone, and plasminogen activator, have also been detected in immunoblots of plant extracts (Doran, 2006). Several strategies have been developed to stabilize recombinant proteins *in planta*, including the use of stabilizing agents, protease gene knockdown, subcellular targeting, fusion proteins, and companion protease inhibitor (Grosse-Holz et al., 2018). Among the attempts, protease inhibitor co-expression has been promising (Benchabane et al., 2008). Therefore, in the future, simultaneous protease inhibitor expression in plants could help to prevent unintended cleavage of recombinant rPA and improve the general productivity of our established rPA expression platform in the future.

### Characterization of Purified Recombinant Reteplase

**Figure 4:**
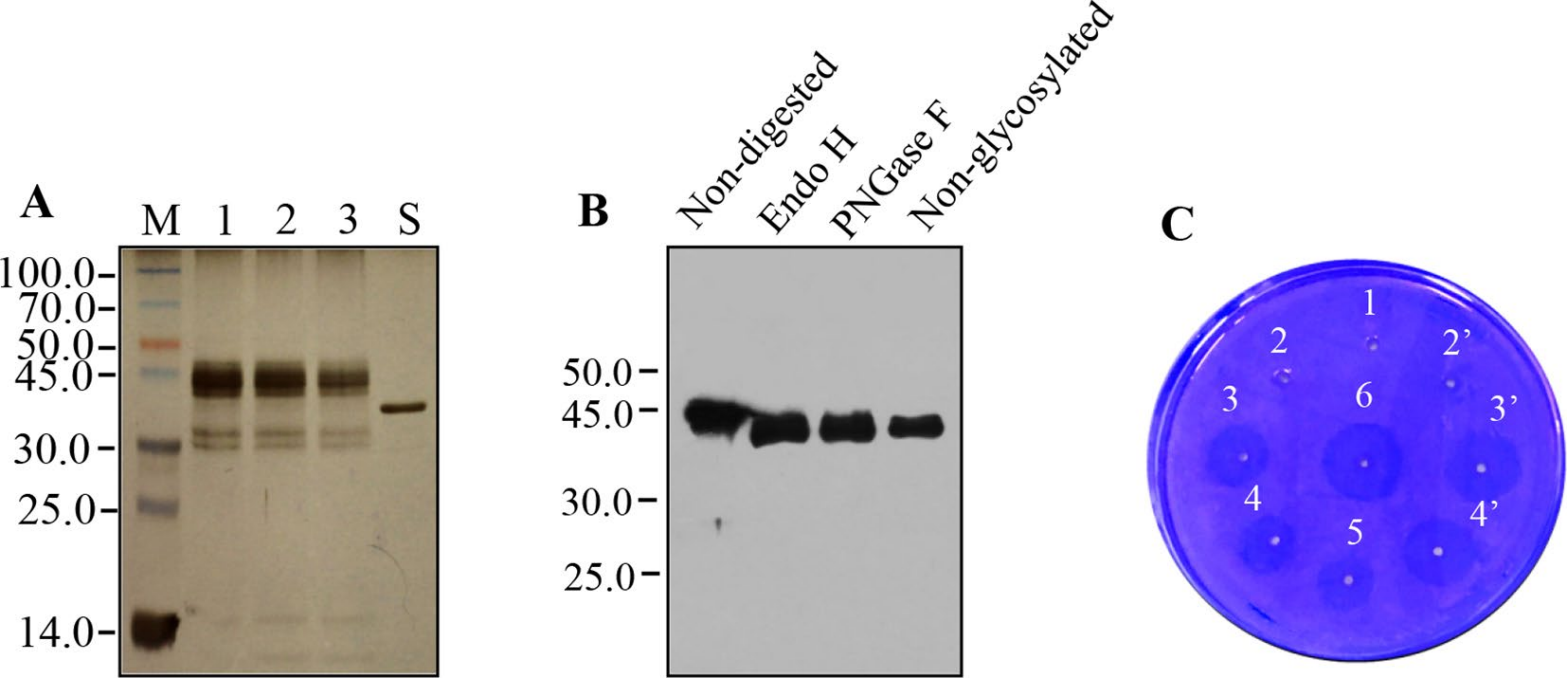
Analysis of recombinant Reteplase (rPA) purified from *N. benthamiana*. **(A)** Silver-stained polyacrylamide gel showing molecular weight and electrophoresis pattern of purified rPA. M, Protein molecular weight markers in kDa; lanes 1, 2, and 3, twofold serial dilution of purified rPA; S, rPA produced in *E. coli* (Roche). **(B)** Glycosylation of rPA produced in *N. benthamiana*. Purified rPA were digested with Endo H or PNGase F and analyzed by western blot using polyclonal antibody against tPA. Non-digested rPA and non-Glycosylated rPA (produced in *E. coli*) were used as a reference as indicated. **(C)** Bioactivity analyses of rPA using fibrin agarose plate assay (FAPA). Samples were spotted onto a plate containing fibrin polymerized by thrombin. The fibrin-plate was incubated at 37°C in a humid incubator overnight. 1, blank control, buffer alone; 2 and 2’, repeats, 0.3 μg of purified rPA with plasminogen activator inhibitor-1 (PAI-1, 1 μg), inhibition of fibrinolytic activity served as negative control; 3 and 3’, repeats, 0.3 μg/ml of purified rPA without PAI-1; 4 and 4’, repeats, 0.15 μg/ml of purified rPA without PAI-1; 5, 0.075 μg/ml of purified rPA without PAI-1; 6, 0.3 μg/ml of rPA produced in *E. coli*, positive control.

Silver-stained polyacrylamide gel analysis of purified recombinant rPA showed abundant diffuse bands at positions ranging from 41.0 to 45.0 kDa and four other faint bands at positions around 30.0 and 14.0 kDa (**Figure 4A**, lanes 1, 2, and 3). *E. coli*-derived rPA showed a band with position corresponding to 39.0 kDa (**Figure 4A**, lane S). This size inconsistency between plant- and *E. coli*-derived rPA is most probably due to the presence or absence of N-glycosylation. The highly diffused bands may correspond to different forms of plant-derived rPA because rPA has two consensus N-glycosylation sites. As already seen in immunoblots (**Figure 3C**), the four other faint bands, two alongside 30.0 kDa and two around 14.0 kDa, likely represent cleavage products of intact rPA. Other proteins were barely visible in silver-stained PAGE. This demonstrates that the purity of plant-derived rPA is comparable with that of an *E. coli*-derived rPA (>90%) control, and the purification procedure *via* Strep-tag II is more appropriate to the needs of rPA purification from the crude plant leaf extracts.

The rPA contains two potential N-glycosylation sites at positions Asp-184 and -448. Therefore, the electrophoretic microheterogeneity of recombinant rPA (**Figure 4A**) urged us to explore whether the rPA that was accumulated in ER of agro-infiltrated *N. benthamiana* leaves was glycosylated. PNGase F and Endo H are the most commonly used endoglycosidase that was generally employed to determine the presence of glycans and their structure of glycosylated proteins (Roth et al., 2012). The PNGase F can remove almost all glycan chain from proteins that are modified by N-linked glycosylation. Endo H can specifically cleave high-mannose N-glycans added in the ER but not complex N-glycans typically found in glycoproteins that reach the Golgi apparatus. Hence, purified rPA was digested with Endo H and PNGase F glycosylases, followed by Western blot analysis using rPA antibodies. *E. coli*-derived rPA was used as unglycosylated control. After treatment with Endo H or PNGase F, a band with a position corresponding to 39.0-kDa rPA appeared (**Figure 4B**), which is identical with that expected for the *E. coli*-derived rPA. The absence of the cleavage fragments, which were present in the previous Western blot analysis, is largely due to the shorter exposure time and lower amount. Nevertheless, the result showed that the plant-produced rPA can be cleaved by both tested glycosidase. It is likely that this plant-derived rPA is glycosylated with high-mannose-type glycans.

Finally, the *in vitro* biological activity of plant-derived rPA was evaluated by fibrin agarose plate assay and compared with *E. coli*-derived rPA. Clearance zones were observed in both treatments of plant-derived and *E. coli*-produced rPA, whereas no such activity was seen in buffer control or plant-derived rPA mixed with plasminogen activator inhibitor-1 (**Figure 4C**). This indicates that the plant-derived rPA was bioactive and has the ability to convert the inactive plasminogen to an active plasmin. The diameter of clearance zones correlated with the concentration of plant-derived rPA in the agarose-fibrin plate (**Figure 4C**), suggesting that the *in vitro* fibrinolysis activity of rPA functions in a dose-dependent manner. Notably, plant-derived and *E. coli*-derived rPA showed similar size of clearance zones when the same dosage was given, indicating that the fibrinolysis activity of plant-derived rPA is functionally comparable with that of commercial *E. coli*-derived rPA. In addition, the presence of glycan side chains in the structure of rPA is not necessary for its function (Mohammadi et al., 2019). That may explain why there is no effect of glycosylation on *in vitro* biological activity of rPA.

## Conclusion

Here, we have evaluated a high-level transient expression system for producing recombinant rPA in tobacco plants by using plant viral amplicon-based expression vector. By comparative analyses of the effects of codon optimization, subcellular targeting, and the position of Strep-tag II on the expression of recombinant rPA, an optimized system was generated that produced up to approximately 50 mg of biologically active rPA from per kilogram fresh weight leaf biomass in less than 1 week. To the best of our knowledge, the yield of bioactive rPA produced with this viral RNA-based expression system exceeds most of that obtained with other plant expression systems reported so far. Furthermore, the purification procedure *via* Strep-tag II offers a facile and rapid strategy for one-step purification of recombinant rPA from *N. benthaniana* leaf extracts. The purified rPA is glycosylated with high-mannose-type glycans, and the *in vitro* fibrinolysis activity is equivalent to the commercially sourced *E. coli*-derived rPA. Accordingly, we propose that the system described here represents an easier and faster alternative to the *E. coli* expression platform, which requires labor-intensive downstream processing. In addition, it also provides a convenient expression platform for further improving clinical efficiency and pharmacokinetic properties of rPA by a genetically engineering approach.

## Data Availability Statement

The raw data supporting the conclusions of this manuscript will be made available by the authors, without undue reservation, to any qualified researcher.

## Author Contributions

SW conceived, designed, and supervised the study, discussed the results, and wrote the manuscript. TM and ZL performed the experiments, analyzed the data, and wrote the manuscript. All authors approved the final manuscript.

## Funding

This work was supported by the National Natural Science Foundation of China (project nos. 31660037 and 31160032).

## Conflict of Interest

The authors declare that the research was conducted in the absence of any commercial or financial relationships that could be construed as a potential conflict of interest.
